# MD-1, a poly herbal formulation indicated in diabetes mellitus ameliorates glucose uptake and inhibits adipogenesis – an in vitro study

**DOI:** 10.1186/s12906-018-2177-x

**Published:** 2018-04-02

**Authors:** Srivani Telapolu, Mangathayaru Kalachavedu, Alan Mathew Punnoose, Dwarakanath Bilikere

**Affiliations:** 1 SRU Center for Indian Systems of Medicine - Quality Assurance and Standardization, Central Research Facility, Sri Ramachandra University, Porur, Chennai India; 2 Faculty of Pharmacy, Sri Ramachandra University, Porur, Chennai India; 3 Centre for Regenerative Medicine and Stem Cell Research, Central Research Facility, Sri Ramachandra University, Porur, Chennai India; 4 Present address: Shanghai Proton and Heavy Ion Center, Pudong, Shanghai China

**Keywords:** Poly herbal formulation, Standardization, Diabetes mellitus, Glucose uptake, Adipogenesis, Peroxisome proliferator activated receptor gamma, Glucose transporter 4

## Abstract

**Background:**

Type 2 Diabetes (T2D) is a polygenic disease requiring a multipronged therapeutic approach. In the current scenario, the use of polyherbals is increasing among the diabetics. MD-1, a poly herbal formulation is constituted as a mixture of six popular anti diabetic herbs, used in the management of Diabetes mellitus (DM). The physicochemical, biochemical and in vitro efficacy studies have been carried out to ascertain the possible mechanisms underlying the anti-diabetic action of MD-1.

**Methods:**

MD-1 was evaluated for residual toxins as per Ayurvedic Pharmacoepia of India (API) procedures. The hydro alcoholic extract of the formulation (HAEF) was evaluated for anti oxidant activity against 2, 2-diphenyl-1-picrylhydrazil (DPPH) and nitric oxide radicals in vitro. The effect of HAEF on carbohydrate digestive enzymes α-glucosidase and α-amylase was studied using biochemical assays. HAEF was studied for its glucose lowering potential in L6 myotubes and 3T3L1 preadipocytes, using 2-deoxy-D-[1-^3^H] glucose (2-DG) uptake assay. Effect of MD-1 on adipogenesis was evaluated in 3T3L1 adipocytes using oil O red staining. The effect of HAEF on mRNA expression of peroxisome proliferator activated receptor gamma (PPARγ) and glucose transporter 4 (GLUT4) in 3T3L1 adiocytes was investigated by reverse transcriptase polymerase chain reaction (RT-PCR). Statistical analysis was performed by student t-test, ANOVA.

**Results:**

Residual toxins present within the API limits and HAEF demonstrated strong antioxidant potential and significantly inhibited the α-glucosidase (IC_50_ 63.6 ± 0.46 μg/mL) and α-amylase (IC_50_ 242.81 ± 1.26 μg/mL) activity. HAEF significantly (*p* < 0.05) enhanced the insulin stimulated glucose uptake in both the cell lines studied. Unlike standard pioglitazone (PGZ), HAEF modulated the mRNA expression of PPARγ and GLUT4 (*p* < 0.0001) in 3T3L1 adipocytes, without inducing adipogenesis.

**Conclusion:**

Physicochemical parameters established in the study may serve as reference standards in regular quality control. Absence of residual toxins underpins the safety. The enhanced glucose uptake and favorable modulation of insulin sensitivity through a plausible weak PPARγ agonism is similar to the distinct PPARγ activation pattern of several reported natural compound agonists. The differential binding modes of such dynamic combinatorial ligands within the formulation unlike synthetic ligands like thiozolidinediones (TZD) can be linked to the safe mitigation of diabetic complications by MD-1.

## Background

DM is a chronic metabolic disorder resulting from either insulin insufficiency or insulin dysfunction [1]. Global prevalence of DM is on the rise especially in developing nations. In India, the number of people suffering from DM has increased from 32 million in 2000 to 63 million in 2013 and is estimated to rise up to 101 million by 2030 [2]. Diabetes is a polygenic disease with the involvement of oxidative stress and requires a multipronged therapeutic approach [3]. There is a global resurgence of herbal drug usage with World health organization (WHO) promoting their amalgamation into the main stream medicine. Due to complex constitution, poly herbals appear to work in a dynamic way to produce better therapeutic activity by interacting with multiple receptor targets. In the Indian systems of medicine, traditional practitioners formulate and dispense their own formulations with combinations of rejuvenating herbs called rasayana drugs. Implicit presence of residual toxins [4], personnel errors in plant collection, seasonal variations, ecotypic, genotypic and chemotypic changes are attributed to the failure of new herbal formulae from producing the intended therapeutic effects [5]. Therefore, the development of standardized, safe and effective herbal formulations with better understanding of their molecular mechanisms offer alternatives in drug discovery for T2D [6].

MD-1 is a poly herbal health supplement indicated in the management of diabetes. It is formulated as a hard gelatin capsule containing a dried and powdered mixture of six medicinal herbs reported for anti diabetic activity. This new herbal formula contains *Phyllanthus amarus* (aerial parts) [7], *Tinospora cordifolia (*stems and leaves) [8]*, Emblica officinalis* (fruits) [9] and *Eugenia jambolana* (fruits) [10], *Gymnema sylvestre* (leaves) [11] and *Cassia auriculata* (flowers) [12]. The composition of herbs is given Table 1. MD-1 is being used in clinical practice at a dose of 500 mg per day in pre diabetes and 1000 mg per day in T2D along with the regular prescription of medicine. It was observed that MD-1 supplementation reduced the risk of disease progression and regression from prediabetes to normal glucose regulation.

**Table 1:** Composition of MD-1

Name of the Herb	Parts used	Quantity per capsule
*Phyllanthus amarus*	Aerial parts	100 mg
*Tinospora cordifolia*	Stems and roots	75 mg
*Emblica officinalis*	Fruits	75 mg
*Eugenia jambolana*	Fruits	75 mg
*Gymnema sylvestre*	Leaves	100 mg
*Cassia auriculata*	Flowers	75 mg

The present work evaluated the quality of MD-1 as per Ayurvedic Pharmacoepia of India (API) procedures. Further the formulation was biochemically evaluated in vitro for antioxidant and anti diabetic activity. Effect of MD-1 on glucose uptake was studied in L6 myotubes and 3T3L1 mouse fibroblast cell lines. Effect of MD-1 on lipid metabolism was assessed in 3T3L1 cells. The mRNA expression of PPARγ and its target gene GLUT4 was investigated in 3T3L1 cell lines to understand the mechanism of action involved.

## Methods

### Materials

MD-1 capsules (3 different batches) were purchased from Isha Arogya, Chennai, Tamilnadu. The capsule contents were collected from twenty capsules of each batch and stored in a amber colour container at room temperature. Calibration standards for heavy metal analysis (Cd, Pb, As, Hg) were purchased from Merck, Germany. 2-Deoxy- D-[1-3H] glucose was purchased from PerkinElmer, USA. Insulin, dexamethasone (DEX), Iso Butyl Methyl Xanthine (IBMX) were purchased from Sigma Aldrich, USA. Pioglitazone (PGZ) was a kind gift from Dr. Reddys Laboratories, India. All other chemicals and solvents were of analytical grade obtained from SISCO Research Laboratories Pvt Ltd. India.

### Physico chemical evaluation

The MD-1 herbal mixture was evaluated for organoleptic properties [13], pH, ash values, extractive values, foreign matter and moisture content as per standard protocols given by API [14].

### Determination of toxic contaminants

The residual analysis was performed to estimate the toxic contaminants like heavy metals, pesticide residues, aflatoxins and microbial load.

### Heavy metal analysis

Heavy metal analysis was performed according to the API guidelines [15]. Sample digestion was carried out by acid digestion method using nitric acid to determine the heavy metal content. After digestion, the samples were analyzed in Atomic Absorption Spectrometer (PERKIN ELMER AAS- 200) for Lead (Pb), Arsenic (As), Cadmium (Cd) and Mercury (Hg). The mercury vapour atomization and hybrid vapour generation attachments were used for AAS analysis of Hg and As respectively. The standards of Pb, As, Cd and Hg (Merck, Germany) were used for the the development of calibration curves.

### Pesticide analysis

The samples were prepared by QuEChERS method to determine the pesticide content [16]. The sample was homogenized by blender and extracted with 1% acetic acid buffer. Internal standards were added to the extraction mixture and shaken vigorously for 1 min. After centrifugation at 1500 g for 1 min, the supernatant was collected and treated with magnesium sulphate for clean up. After 30 s, the supernatant was collected by centrifugation at 1500 g for 1 min and used for analysis. The quantitative estimation of organo chlorine, organo phosphorous and pyrethroids was carried out using GC/MS analysis in a DB-5 (30 m × 0.25 mm × 0.25 μm) capillary column (Agilent 7000 Triple Quad GC/MS, USA) [17]. Recovery studies with purified compounds indicated that the overall recovery value was 85%.

### Aflatoxin determination

The samples were extracted using methanol: water (17:3) mixture to estimate the aflatoxin content. The filtrate was treated with zinc acetate: aluminium chloride reagent to avoid the interfering pigments. Further, clean up was carried out as per the procedures given by API [15]. Aflatoxins were estimated using Waters Alliance 2695 HPLC instrument using a Luna C18 column (Phenomenex) of dimensions 4.6 × 150 mm × 5 μ coupled with Waters 2475 fluorescence detector containing Cobra cell [17]. The excitation wavelength and the emission wavelength for fluorescent detection were set at 362 nm and 455 nm, respectively. The calibration standards were procured from Sigma Aldrich.

### Microbial load analysis

Microbial analysis was carried out as per API guidelines [15]. The sample was suspended in 0.1% *w*/*v* of polysorbate 80 and kept on a mechanical shaker for few minutes. The dilution was 1:100 or 10^− 2^ from, which 1 mL was transferred to 9 mL of sterilized distilled water to make a 1:1000 dilution and this procedure was repeated up to 10^− 6^ dilution. Each 0.1 mL of serially diluted sample was inoculated to the sterile plates containing casein soya bean digest agar and plates were incubated at 37 °C for 24 h to evaluate the bacterial count. Further, the samples were incubated in Sabouraud dextrose agar plates at room temperature for 5 days to evaluate the fungal load. The bacterial and fungal colonies were counted using a colony counter. The presence of specific micro-organisms *Escherichia coli, Salmonella ebony, Pseudomonas aeruginosa* and *Staphylococcus aureus* was identified using biochemical tests such as indole test, triple sugar test, oxidase test and coagulase test, respectively.

### Preparation of extract

The sample (5 g) was extracted thrice with 70% methanol (3Χ25 mL) by cold maceration for 24 h. The filtrate was concentrated in a rotary vacuum evaporator (Superfit, India) at 60 °C (yield 10.32 %*W*/W; 51.6 mg/capsule).

### Phyto chemical analysis

HAEF was dissolved in ethanol: water (7:3) mixture to obtain 1% *w*/*v* solution and used for qualitative [18] and quantitative estimation of phytochemicals. The total phenol content was estimated using Folin-ciocalteau method [19] and calculated as gallic acid equivalents. The total flavonoid content was determined using AlCl_3_ method [20] and calculated as quercetin equivalents.

### In vitro efficacy studies

#### Antioxidant activity

##### 2, 2-Diphenyl-1-picrylhydrazil (DPPH) radical scavenging activity [21]

The HAEF (0.12–125 μg in 20 μL of DMSO) was incubated with (180 μl) 50 μM DPPH at 37 °C for 30 min in dark to evaluate the antioxidant activity against DPPH radicals. The absorbance was measured at 517 nm using multi mode reader (PerkinElmer Enspire, USA). The percentage DPPH radical scavenging activity was calculated against solvent control. Ascorbic acid was used as a positive control.

##### Nitric oxide scavenging activity [22]

The HAEF (0.12–125 μg/mL in DMSO) was incubated with 10 mM sodium nitroprusside (300 μL) in phosphate buffer (pH 7.4) at 30 °C for 2 h to evaluate the anti oxidant activity against nitric oxide radicals. Following the incubation, 500 μL of Griess reagent (1% sulfanilamide, 2% H_3_PO_4_ and 0.1% N-(1-naphthyl) ethylenediamine dihydro chloride) was added to the reaction mixture. The absorbance of the chromophore (purple azo dye) formed during the diazotisation of nitrite ions with sulphanilamide and subsequent coupling with naphthyl ethylene diamine dihydro chloride was measured at 546 nm. The percentage NO radical scavenging activity was calculated relative to the solvent control. Ascorbic acid was used as a positive control.

##### Effect on advanced glycation end product (AGE) formation [23]

The HAEF (0.12–125 μg/mL in DMSO) was incubated with a reaction mixture containing 10 mg/mL bovine serum albumin in 50 mM sodium phosphate buffer (pH 7.4), 0.02% sodium benzoate in 0.2 M fructose and 0.2 M glucose for 7 days to evaluate the effect on AGEs formation. Following the incubation, the fluorescence was measured at excitation and emission wavelengths of 350 nm and 450 nm, respectively using multi mode reader (PerkinElmer Enspire, USA). The percentage inhibition was calculated with respect to the solvent control. Amino guanidine was used as a positive control.

##### Effect on α-glucosidase (EC 3.2.1.20) activity [24]

In order to determine the effect of HAEF on α-glucosidase activity, the enzyme solution (10 mg/mL) was pre-incubated with 200 μL of HAEF (1.95–1000 μg/mL in DMSO) for 5 min. Then, 37 mM sucrose (200 μL) was added to initiate the reaction. The reaction was terminated after incubation at 37 °C for 15 min by heating at 90–100 °C. The liberated glucose was estimated using the commercial kit (Accurex, India). The percentage inhibition of α-glucosidase activity was calculated with respect to the solvent control. Acarbose was used as a positive control.

##### Effect on α –amylase (EC 3.2.1.1) activity [25]

In order to determine the effect of HAEF on α- amylase activity, 200 μL of 10 μM diastase solution was incubated with a reaction mixture containing 200 μL of HAEF (1.95–1000 μg/mL in DMSO), 250 μL of 0.02 M sodium phosphate buffer (pH 6.9 with 0.006 M NaCl) and 250 μL of 0.2% starch solution at 25 °C for 10 min. After incubation, the reaction was terminated by boiling at 90–100 °C for 1 min. Then, dinitro salicylic acid (DNS) colour reagent (500 μL) was added and the tubes were incubated in boiling water bath for 5 min. After cooling to the room temperature, the absorbance was measured at 540 nm using multi mode reader (PerkinElmer Enspire, USA). The percentage inhibition of α-glucosidase activity was calculated with respect to the solvent control. Acarbose was used as a positive control.

### Cell culture

L6 myocytes and 3T3L1 preadipocytes were procured from the National Centre for Cell Science, Pune and maintained in Dulbeccos Modified Eagle Medium (DMEM) containing high glucose with 10% fetal bovine serum (FBS) and supplemented with penicillin (5 units/mL) and streptomycin (5 μg/mL) in 5% CO_2_ incubator at 37 °C. HAEF stock solution (5 mg/mL) was prepared using 10% DMSO and dilutions were prepared in DMEM medium. Addition of HAEF did not affect the pH of culture media (7.35–7.45) as monitored by the color change with the phenol red indicator included in the medium as well as measurements made using pH meter (Mettler Toledo, India).

### Cytotoxicity

The cytotoxicity of HAEF in both cell lines was evaluated by 3-(4, 5-dimethylthiazol-2-yl)-2, 5-diphenyltetrazolium bromide (MTT) colorimetric assay [26]. Briefly, the cells were seeded in a 96-well plate at a density of 5× 10^3^ cells/well in DMEM high glucose media with 10% FBS. After 24 h, the cells were treated with HAEF at different concentrations in serum free media for 24 h and 7 days in L6, 3T3L1 cells respectively. Post incubation, the MTT assay was carried out and the absorbance was measured at 570 nm in multi mode reader (Perkin Elmer, USA). HAEF was found to be safe up to the concentration of 250 μg/mL (Fig. 4a, b).

### Effect of HAEF on glucose uptake

Effect of HAEF on glucose uptake was studied in L6 myotubes and 3T3L1 adipocytes as per the reported method [27] with slight modifications. Briefly, post confluence, L6 cells were transferred to DMEM media with 2% FBS for 4 days to induce myotube formation. After two days of confluence, the 3T3L1 pre adipocytes were switched to differentiation medium containing 0.25 μM DEX, 0.5 mM IBMX and 1 mg/L of insulin in DMEM medium with 10% FCS to induce differentiation (day 0). After 72 h of induction, the media was replaced with maintenance media containing 1 mg/mL insulin for 48 h (day 5). The media was subsequently replaced again with fresh culture media (DMEM with 10% FBS), after 2 days. After differentiation, the cells were serum starved for 5 h and incubated with HAEF for 24 h. Cells were stimulated with 100 nM insulin or left untreated for 30 min. After incubation with 0.5 μCi/mL 2-DG for 15 min, cells were washed with phosphate buffered saline (PBS) and lysed with 0.1% sodium dodecyl sulphate (SDS). The cell lysates were transferred to scintillation cocktail to measure radioactivity using liquid scintillation counter (Perkin Elmer, USA). Results were expressed as percentage glucose uptake relative to their respective controls. PGZ was used as a standard drug.

### Effect of HAEF on triglyceride accumulation [28]

3T3L1 pre adipocytes were cultured in 48 well plate. After two days of confluence, differentiation was induced as mentioned above. Pre adipocytes were maintained with fresh growth media every other day throughout the experiment. The cells were treated with HAEF through day 0 to day 7 to study the effect of HAEF on differentiation. Oil O red staining was performed to visualize the triglyceride accumulation and microscopic images were captured using Nikon digital camera. The stain was further collected into isopropanol and quantified using multi plate reader (Perkin Elmer, USA) at 492 nm.

### mRNA expression of PPARγ and glut 4 in 3T3L1 cell line

The 3T3L1 cells were seeded in a six well plate at a density of 1× 10^6^ cells and cultured in differentiation medium with HAEF to study the effect of HAEF on the mRNA expression of adipocyte specific genes. Total RNA was isolated using TRIZOL reagent and quantified using biophotometer (Eppendorf, Germany). About 1 μg of total RNA was converted into cDNA using Thermoscientific verso cDNA synthesis kit, according to the manufacturer’s instructions. Reverse transcriptase polymerase chain reaction (RT –PCR) was performed using the Amplicon master mix as per manufacturer’s protocol. The primer sequences used for PCR analysis were as follows:PPARγ:5’-ACCTGAAGCTCCAAGAATACCA-3′(forward) and 5’-TAAGCTTCAATCGGATGGTTCT-3′(reverse),GLUT4:5’ TGGACCTGTAACTTCATTGTCG-3′(forward) and 5’-TCTGTACTGGGTTTCACCTCCT-3′(reverse), GAPDH:5’-ACCACAGTCCATGCCATC-3′(forward) and 5’-TCCACCACCCTGTTGCTG-3′(reverse). The reaction mixture was subjected to denaturation, annealing and extension at 95 °C for 30 s, 57 °C for 30 s, 72 °C for 1 min for 35 cycles in Master cycler gradient (Eppendorf, Germany). PCR products were analysed by electrophoresis in 1% agarose gel at 80 V and the fragments were visualized by safe dye staining. Photo documentation was performed using Quantity One 1-D Analysis Software. The gene expression was shown as ratio of densitometry value of target mRNA to that of GAPDH.

### Statistical analysis

Statistical analysis was performed using Graph pad prism software version 6. (Graph pad software,USA). Unpaired t test was used to compare the difference between the test and standard. One way ANOVA followed by post hoc Dunnet’s test was used to compare the means between different groups. Two-way repeated measures ANOVA with Bonferroni correction was performed to compare all time course data within the groups. *P* < 0.05 was considered to be statistically significant.

## Results

### Physicochemical and phytochemical analysis

Physicochemical parameters serve as an important reference in regular quality control during the scaling up of production. The poly herbal mixture in the commercially procured hard gelatin capsules of MD-1 is of a light green colour, possessing a characteristic odour and bitter taste with moderately fine texture. The physicochemical parameters of the herbal mixture such as ash and extractive values were established with respect to the dry weight of the powder mixture (Table 2). There were no visible signs of mould growth, contamination by insects and other animals in the powder mixture which in turn indicated the absence of foreign organic matter. The total phenolic content was 116 mg/g of gallic acid equivalents, whereas the flavonoid content was 288 mg/g of quercetin equivalent.

**Table 2:** Physicochemical parameters

Parameter	%W/W
Total ash	11.59 ± 1.02
Water soluble ash	3.76 ± 0.15
Acid insoluble ash	2.28 ± 0.54
Water soluble extractive	9.2 ± 0.35
Alcohol soluble extractive	6.56 ± 1.07
Loss on drying	9.55 ± 1.09
pH value	4.12 ± 0.02

The values are expressed as mean ± SEM of triplicate determinations

### Residual analysis

Residual analysis of MD-1 demonstrated the absence of aflatoxins B1, B2, G1, G2 (Table 3) and pesticide classes, such as organophosphorus, organochlorine and pyrethroids (Table 4). Heavy metals Cd, Hg, As and Pb were present below the specified limits by Ayurvedic Pharmacopoeia of India (Table 5). The microbial profile of MD-1 was found to be satisfactory with total bacterial count of 4.27 × 10^4^ CFU/g. There was no fungal contamination and the pathogenic microorganisms like *Salmonella, Pseudomonas, Staphylococcus* and *E. coli* were not present (Table 6).

**Table 3:** Aflatoxin analysis

Aflatoxin	Observation	Acceptable limit
B1	ND (LOD 0.5 ppb)	5 ppb (B1 + B2 + G1 + G2)
B2	ND (LOD 0.5 ppb)	
G1	ND (LOD 0.5 ppb)	
G2	ND (LOD 0.5 ppb)	

The values are expressed as mean ± SEM of triplicate determinations

**Table 4:** Pesticide residue analysis

Pesticide residue	Results	Acceptable limit
*Organochlorine pesticides*		
DDT	ND (LOD 0.01 ppm)	1 ppm
Endosulphan alpha	ND (LOD 0.01 ppm)	3 ppm (sum of isomers)
Endosulphan beta	ND (LOD 0.01 ppm)	
*Organophosphorous pesticides*		
Chlorpyrifos	ND (0.05 ppm)	0.2 ppm
*Pyrethroids*		
Cypermethrin	ND (LOD 0.1 ppm)	1 ppm

The values are expressed as mean ± SEM of triplicate determinations

**Table 5:** Heavy metal analysis

Element	Results (ppm)	Acceptable limit (ppm)
*As*	ND (LOD 0.05 ppm)	3
*Pb*	0.097 ± 0.01 (LOD 0.05 ppm)	10
*Hg*	0.033 ± 0.002 (LOD 0.01 ppm)	1
*Cd*	0.04 ± 0.001 (LOD 0.01 ppm)	0.3

The values are expressed as mean ± SEM of triplicate determinations

**Table 6:** Microbial analysis

Parameter	Observation	Acceptable limit
Total bacterial count (Cfu/g)	4.27 × 10^4^	10^5^–10^7^/g
Total Mould count (Cfu/g)	Absent	10^3^/g
*E.coli*	Absent	Absent
*Salmonella spp*	Absent	Absent
*Staphylococcus aureus*	Absent	Absent
*Pseudomonas aeroginosa*	Absent	Absent

The values are expressed as mean ± SEM of triplicate determinations

### HAEF demonstrated strong anti oxidant and anti glycation activity

**Fig. 1:**
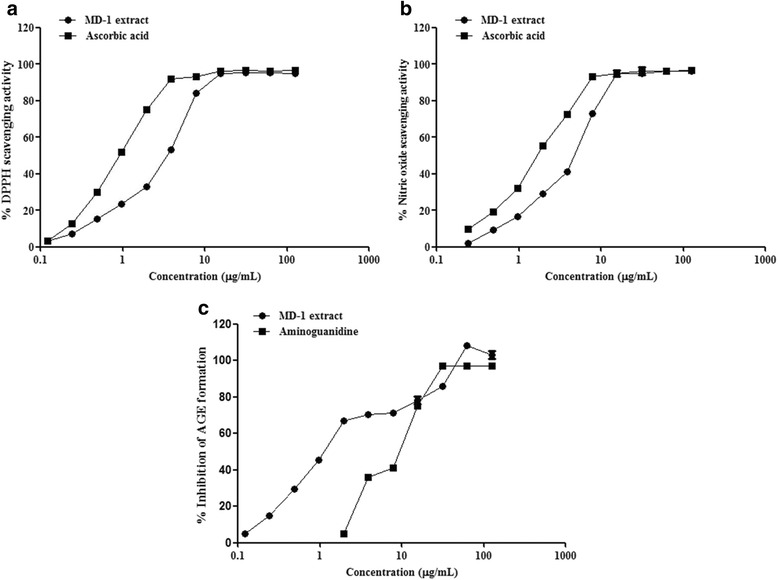
Effect of HAEF on free radical scavenging and Advanced glycation end product formation. Anti oxidant activity of HAEF against (**a**) 1,1-Diphenyl-2-picrylhydrazyl (DPPH) radical; (**b**) Nitric oxide scavenging; (**c**) Advanced glycation end product formation. All the values were expressed as mean ± SEM of triplicate determinations

The anti oxidant activity of HAEF was evaluated using DPPH free radical and nitric oxide scavenging assays (Fig. 1a, b). HAEF showed a dose dependent quenching of DPPH and NO radicals with an EC_50_ of 3.3 ± 0.18 μg/mL and 4.46 ± 0.56 μg/mL, respectively whereas the reference standard ascorbic acid demonstrated strong anti oxidant activity with an EC_50_ of 0.84 ± 0.16 μg/mL and 1.8 ± 0.96 μg/mL, respectively. Although the ascorbic acid demonstrated significantly stronger activity than HAEF, it was observed that both of them showed the maximum radical quenching (95%) at 15.62 ± 0.04 μg/mL. In anti glycation assay, HAEF significantly (*p* < 0.05) inhibited the AGEs formation with an IC_50_ 1.03 ± 0.54 μg/mL when compared to the reference standard, amino guanidine (IC_50_ 7.09 ± 0.16 μg/mL) (~ 7 fold higher) (Fig. 1c).

### HAEF inhibited the enzymes regulating carbohydrate digestion

**Fig. 2:**
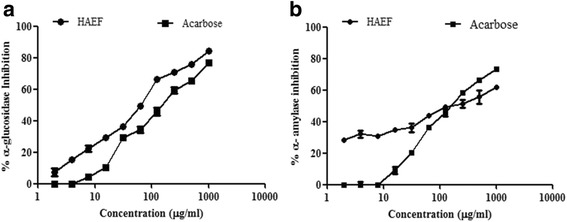
Effect of HAEF on the carbohydrate digestive enzymes. Effect of HAEF on carbohydrate digestive enzymes (**a**) α-Glucosidase; (**b**) α-Amylase. All the values were expressed as mean ± SEM of triplicate determinations. (**a**) *P < 0.05 against acarbose

α- Glucosidase and pancreatic α – amylase are the carbohydrates enzymes, which facilitate the glucose absorption into blood stream by converting poly and disaccharides to monosaccharides [29]. HAEF inhibited α- glucosidase activity in dose dependent manner with an IC_50_ 63.6 ± 0.46 μg/mL, while the reference standard, acarbose showed 50% inhibition at 136.28 ± 1.24 μg/mL only. HAEF showed significantly (*P* < 0.05) stronger inhibition than standard acarbose with maximum of 84.29 ± 1.29% activity at 1000 μg/mL (Fig. 2a). HAEF and standard acarbose demonstrated strong inhibitory action on α – amylase in a dose dependent manner with an IC_50_ 242.81 ± 1.26 μg/mL and 134.41 ± 2.13 μg/mL, respectively (Fig. 2b).

### HAEF improved insulin stimulated glucose uptake in L6 myotubes and 3T3L1 adipocytes

**Fig. 3:**
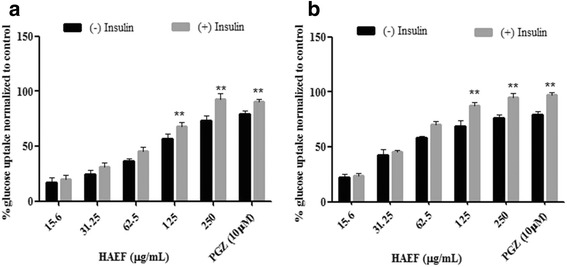
Effect of HAEF on Glucose uptake in L6 myotubes & 3T3L1 adipocytes. Effect of HAEF on glucose uptake on (**a**) L6 myotubes & (**b**) 3T3L1 adipocytes. The cells were treated with different concentrations of HAEF and pioglitazone (PGZ) for 24 h in the presence and absence of insulin. All the data were expressed as means ± S.E.M. of three independent experiments. *P < 0.05 as compared with respective (−) insulin group

**Fig. 4:**
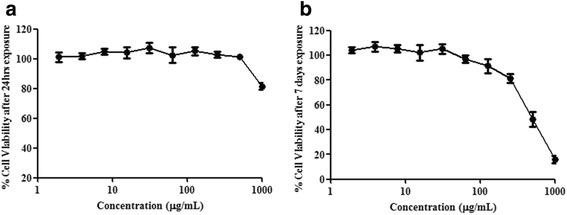
Cytotoxicity assay in L6 myotubes & 3T3L1 adipocytes. Effect of HAEF on cell viability of (**a**) L6 myotubes after 24 h exposure (**b**) 3T3L1 adipocytes after 7 days exposure. All the data were expressed as means ± S.E.M. of three independent experiments

A cell based assay was carried out using L6 myoblasts and 3T3L1 preadipocytes to identify MD-1 that could stimulate the glucose uptake like insulin. Both the cell lines were differentiated into L6 myotube and 3T3L1 adipocyte phenotype which are efficient models to measure glucose uptake [30]. The differentiated cells were incubated with HAEF and after incubation, the cells were assessed for 2-deoxy -D- [1-^3^H] glucose uptake. HAEF demonstrated dose dependent increase in glucose uptake relative to untreated cells in both the cell lines. The optimum dose of 250 μg/mL demonstrated more than 70% uptake at basal condition and 90% uptake in insulin stimulated conditions which are much similar to reference standard PGZ (Fig. 3a, b). At higher concentrations insulin stimulated glucose uptake was significantly (*p* < 0.05) increased when compared to the basal uptake. Additionally HAEF was found to be non toxic even at higher concentrations (Fig. 4a, b).

### HAEF inhibited lipid accumulation and down regulated the mRNA expression of adipogenic transcription factors

**Fig. 5:**
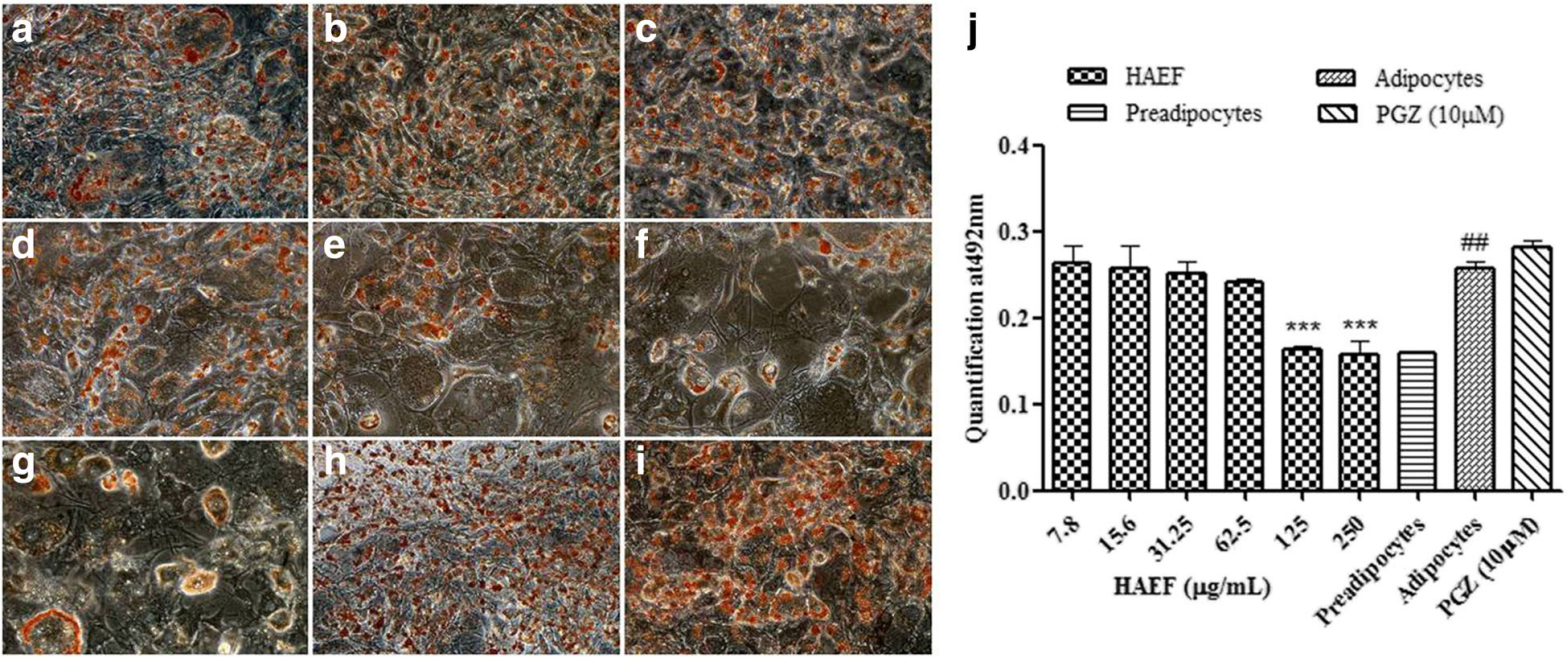
Effect of HAEF on lipid accumulation. Effect of HAEF on lipid accumulation in 3T3L1 preadipocytes after differentiation induction- Oil O red staining and photo microscopic evaluation at 40X **a** -**f** 3T3L1 preadipocytes induced for differentiation in the presence of HAEF at 7.8, 15.6, 31.25, 62.5, 125, 250 μg/ml treated cells; (**g**) Preadipocyte control; (**h**) adipocyte control; (**i**) Pioglitazone (PGZ) at 10 μM; (**j**) Quantification of Oil O Red stain at 492 nm. All the data were expressed as means ± S.E.M. of three independent experiments. ***P < 0.0001 as compared with respect to adipocyte control & ##*P* < 0.001 as compared with respect to preadipocytes

**Fig. 6:**
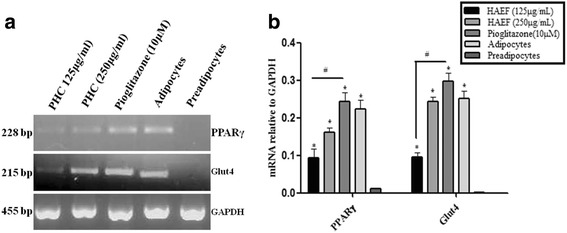
Effect of HAEF on mRNA expression of PPARγ and GLU4. Effect of HAEF on mRNA expression of PPARγ and GLUT4 in 3T3L1 preadipocytes (**a**) RT-PCR (**b**) Quantification of bands using Image J software. All the data were expressed as means ± S.E.M. of three independent experiments. *P < 0.0001 as comparision with pre adipocytes, #*P* < 0.0001 as comparision with pioglitazone

Adipogenesis is a complex process controlled by several harmones, adipokines and growth factors. MD-1 effect on adipogenesis was studied in vitro using 3T3L1 preadipocytes. Post confluence, 3T3L1 preadipocytes were induced for differentiation in the presence of HAEF at different doses. Oil O Red staining was performed to measure intracellular lipid accumulation. HAEF dose dependently reduced the triglyceride accumulation in differentiated adipocytes. Only basal level of lipid accumulation was observed in cells treated with HAEF at a dose of 125 μg/mL and 250 μg/mL (Fig. 5). HAEF was significantly (*p* < 0.0001) inhibited the lipid accumulation when compared to PGZ (used as a negative control), however HAEF exhibited a similar activity to PGZ in glucose uptake assay (Fig. 3b). Therefore for better understanding the mechanism of action, the expression level of genes involved in the glucose and lipid metabolism was studied in 3T3L1 preadipocytes induced for differentiation in the presence of HAEF. HAEF brought about an increased expression of mRNA of PPARγ and Glut 4 when compared to the pre adipocyte control; however, it was lesser than that of PGZ (Fig. 6). Altogether these results are indicating the partial agonistic activity of MD-1 towards PPARγ.

## Discussion

WHO recommends the standardization of HMPs, pending which many herbal formulations remain as herbal supplements which are otherwise part of first line approach in clinical practice [31]. MD-1 is one such formulation that has been in clinical practice for the management of pre diabetes and T2D. In this study MD-1 has been evaluated for its quality according to API standard procedures. Biochemical and in vitro efficacy studies have been carried out to ascertain the possible mechanisms underlying the anti-diabetic action of MD-1.

The physicochemical parameters established for MD-1 may serve as reference standards in regular quality checks. As per WHO, presence of heavy metals, aflatoxins, pesticide residues and pathogenic microorganisms in herbal medicinal products (HMPs) make them non-compliant with the quality requirements of the regulatory authorities [32]. In this context, the absence of toxic residues in MD-1 ensures the quality of starting material/ medicinal herbs which are compliant with WHO guidelines on Good Agricultural and collection practices (GACP).

In diabetes, elevated levels of glucose enhance the gene and protein expression of eNOS and iNOS thereby increased production of NO which leads to oxidative stress [33]. Likewise many of the biochemical pathways associated with hyperglycemia will increase the production of free radicals [34]. The strong antioxidant activity demonstrated by MD-1 would possibly bring about the reduction of oxidative stress in diabetes and prediabetes. The presence of large amounts of poly phenols in HAEF was amply supportive of the observed antioxidant activity [35].

AGEs are proteins or lipids that become glycated as a result of exposure to sugars. Hyper glycaemia, auto-oxidation of glucose, glycation of proteins and anti-oxidant enzymes reportedly contribute to the etiology of oxidative stress in diabetes [36]. The anti glycation activity demonstrated by HAEF indicates that MD-1 supplementation may control the development of associated micro vascular complications in diabetes patients. Literature reports on the glycation protective effect of antioxidants such as vitamin C, poly phenols and flavonoids [37] endorsed the observed activity of the polyphenol rich HAEF in anti oxidant activity, thereby also substantiating its effect on AGEs formation.

Inhibition of carbohydrate enzymes in the intestinal lumen is one of the important therapeutic approaches in DM. Inhibition of these enzymes helps to reduce the post-prandial hyperglycemia in diabetes patients [38]. The inhibition of α-glucosidase and α-amylase enzymes by HAEF is expounded by synergistic action of flavonoids, oleanane and ursane triterpenes which are reportedly present in the constituent herbs of the formulation.

Skeletal muscle and adipose tissue are the primary target site for insulin and play a crucial role in post prandial glucose regulation [39]. Defects in signal transduction pathways and decreased GLUT4 translocation lead to the development of insulin resistance [40]. The enhanced glucose uptake by HAEF is suggesting the beneficial effect of MD-1 supplementation in insulin resistance. Presence of phytochemicals like gallic acid [41], ellagic acid [42], berberine [43] and quercetin [44] with reported activation of AMPK pathway substantiated the observed glucose uptake by MD-1 in the absence of insulin. Reported amelioration of insulin resistance by the constituent herbs [45, 46] lends support to the HAEF mediated 2-DG uptake in the presence of insulin.

Adipogenesis, the cellular differentiation of preadipocytes to adipocytes is described as a cascade of gene expressions regulated by a small set of transcription factors. PPARγ is a member of nuclear receptor super family that regulates the expression of many proteins involved in glucose homeostasis and plays a central role in adipocyte differentiation [47]. PGZ is a synthetic TZD and a PPARγ agonist used in the treatment of DM to improve insulin sensitivity in the target tissues. Being potent adipogenesis inducers in vitro [48], the TZDs reduced blood sugar in vivo despite the fact that their administration was often accompanied by the unpleasant side effect of weight gain and fluid retention [49].

Several medicinal plant derived natural products have been identified as PPARγ modulators and have shown improvement in metabolic parameters in diabetic animal models with reduced drug associated toxicities compared to TZD agonists [50]. Compared to the latter, these natural PPARγ ligands were reported to have different binding modes, thus accounting for the lack of delineation of their metabolic effects to PPARγ activation. Search for such PPARγ agonists that selectively modulate the receptor activity maintaining glucose homeostasis without the adverse effects associated with TZD is a promising approach in diabetes research [51]. In this context, the observed anti adipogenic and insulin sensitizing effect of HAEF could be possibly attributable to mechanisms beyond PPARγ activation. Although HAEF displayed the enhancement of PPARγ and GLUT4 expression, it strongly inhibited the triglyceride accumulation and showed glucose uptake similar to PGZ. A weak PPARγ agonistic activity by HAEF was thus hypothesized, given the fact that majority of identified natural compounds acted as weak agonists with activation pattern distinct from full TZD agonists and more similar to endogenous ligands, such as fatty acids and prostanoids, having weaker activation potential.

The phyto chemical complexity of herbal drugs and evaluation of toxicity of bio-actives over parental extracts are the essential requirements for the development of standardized herbals rather than the less rewarding lead molecule isolation approach. Multi target mode of action, high safety and tolerability of phytotherapeutics offer valuable preventive and therapeutic options in holistic diabetes management. This work has laid the blue print for the quality control that is expected to pave way for use of this formulation as a dietary supplement in DM. The effects of MD-1 on glucose uptake, similar to PGZ, as observed on L6 and 3T3L1 cell lines pointed towards its favorable enhancement of insulin sensitivity. The mechanistic insights of PPARγ modulation by HAEF were suggestive of partial agonistic activity of the poly herbal formulation, possibly due to the presence of combinatorial ligands working in a dynamic way to selectively modulate PPARγ activity.

## Conclusion

The present work has established the safety and efficacy profile of MD-1 an essential regulatory requirement for clinical studies and partially unravelled the plausible mechanisms of action as enhanced glucose uptake and PPARγ agonism linked modulation of insulin sensitivity. Absence of residual toxins, inhibitory action on carbohydrate digestive enzymes and AGEs formation in vitro underpinned its safety and efficacy, respectively. Moreover, the analytical parameters investigated in this study for MD-1 - a clinically used anti diabetic health supplement is expected to pave way for the development of quality control reference protocols for its up-scaled production. Further studies required to completely elucidate the molecular mechanism involved in the anti-diabetic activity of MD-1 as well as its effects on whole-body metabolism in diabetic animal models to unequivocally establish its utility in the management of diabetes.

## Abbreviations

2-DG: 2-deoxy-D-[1-^3^H] glucose
API: Ayurvedic Pharmacopoeia of India
DEX: Dexamethasone
DM: Diabetes mellitus
DMEM: Dulbeccos Modified Eagle Medium
DPPH: 2,2-diphenyl-1-picrylhydrazil
GLUT4: Glucose transporter 4
HAEF: Hydro alcoholic extract of the formulation
HMPs: Herbal medicinal products
IBMX: Iso butyl methyl xanthine
MTT: 3-(4,5-dimethylthiazol-2-yl)-2,5-diphenyltetrazolium bromide
PGZ: Pioglitazone
PPARγ: Peroxisome proliferator activated receptor gamma
T2D: Type 2 diabetes
TZD: Thizolidinedione
